# Megakaryocytes as the Regulator of the Hematopoietic Vascular Niche

**DOI:** 10.3389/fonc.2022.912060

**Published:** 2022-06-22

**Authors:** Huichun Zhan, Kenneth Kaushansky

**Affiliations:** ^1^ Department of Medicine, Stony Brook School of Medicine, Stony Brook, NY, United States; ^2^ Medical Service, Northport Veterans Affairs (VA) Medical Center, Northport, NY, United States

**Keywords:** megakaryocyte (MK), vascular niche, hematopoietic stem cell (HSC), myeloproliferative neoplasms, hematopoietic microenvironment

## Abstract

Megakaryocytes (MKs) are important components of the hematopoietic niche. Compared to the non-hematopoietic niche cells, MKs serving as part of the hematopoietic niche provides a mechanism for feedback regulation of hematopoietic stem cells (HSCs), in which HSC progeny (MKs) can modulate HSC adaptation to hematopoietic demands during both steady-state and stress hematopoiesis. MKs are often located adjacent to marrow sinusoids. Considering that most HSCs reside close to a marrow vascular sinusoid, as do MKs, the interactions between MKs and vascular endothelial cells are positioned to play important roles in modulating HSC function, and by extrapolation, might be dysregulated in various disease states. In this review, we discuss the interactions between MKs and the vascular niche in both normal and neoplastic hematopoiesis.

## Introduction

Hematopoietic stem cells (HSCs) reside in a specified microenvironment, or the “stem cell niche”. Technical advancements in imaging HSCs in the marrow cavity, coupled with a series of elegant functional studies in murine models, have identified a number of HSC niche cells (e.g., perivascular stromal cells, endothelial cells, nerve cells) that provide the secreted factors and cell-surface molecules essential for HSC maintenance and function (1–5). It has become increasingly evident that the cellular niche components exist in close proximity to one another, and that the interaction between these cells contributes to the resilience and function of HSCs. Megakaryocytes (MK) are rare polyploid marrow cells that give rise to blood platelets. Recent evidence has also implicated MKs in regulating HSC quiescence and proliferation during both steady-state and stress hematopoiesis, mediated by the many cytokines and extracellular matrix components produced by these cells (6–12). In addition, MKs express many inflammatory and immunologic surface markers and signaling molecules and may participate in pathogen surveillance and immune response (13–18). MKs are often located adjacent to marrow sinusoids, a “geography” required for the cells to issue platelets directly into the sinusoidal vascular lumen (19, 20). Vascular endothelial cells (ECs) are also an essential component of the hematopoietic niche with most HSCs found adjacent to a marrow sinusoid (the “vascular niche”) (3, 21–24). Considering that MKs, sinusoids, and HSCs are closely located to each other, the interactions between MKs and ECs are positioned to play an important role in modulating HSC function. Few studies have examined the role of MKs in the regulation of stem cell vascular niche function, despite MKs representing a major source of both proangiogenic and antiangiogenic factors in the marrow. Here, we review the interactions between MKs and the vascular niche in both normal and neoplastic hematopoiesis.

## Megakaryocytes Are an Important Component of the Hematopoietic Niche

Recent evidence has implicated MKs in regulating HSC quiescence and proliferation during both steady-state and stress hematopoiesis (6–12). For example, MKs produce TGFβ1 and CXCL4 to promote HSC quiescence *in vivo (* 8, 9); during stress hematopoiesis (e.g., after chemotherapy), the production of TGFβ1 and CXCL4 by MKs surrounding clusters of myeloid progenitor cells may help re-establish HSC quiescence (25). MKs can also promote HSC niche remodeling (e.g., osteoblast expansion) following radiation injury (26, 27) and activate HSC proliferation under stress by synthesizing FGF1 and IGF-1 (6–8). Spatial positioning of HSCs next to MKs not only regulates HSC activity, but also affects HSC developmental potential — HSCs located adjacent to MKs are more platelet- and myeloid-biased compared to HSCs enriched in other regions of the marrow (e.g., the arteriolar niche), and MK depletion can reprogram these myeloid-biased HSCs to a lineage-balanced HSCs (28). Taken together, these studies indicate that the functional effects of MKs in the HSC niche are context-dependent and can be tailored based on demands. Compared to the non-hematopoietic niche cells (e.g., endothelial cells, mesenchymal stromal cells), the MK niche provides a mechanism for a feedback regulation of HSCs by their own progeny. The adaptation of the MK niche function to various hematopoietic demands suggests that HSC and its niche can affect each other, which is critical for the resilience and function of the hematopoietic system.

## The Interactions Between Vascular Endothelial Cells and Megakaryocytes in the Hematopoietic Niche

MKs are the largest cells in the marrow with a mean diameter of ~20-50um, with their cell sizes correlating with their degree of maturation (19, 29). MKs are mostly sessile, exhibiting minimal migration in a highly crowded environment (19, 29). ~70% of marrow MKs are intimately associated with the sinusoids, a “geography” required for the cells to issue platelets by the forces generated by flowing sinusoidal blood (19, 20, 30). Ultrastructural observations (31) and intravital imaging (19, 29) revealed that these MKs extend both perivascular pseudopodia to anchor the cells to endothelium, and long transendothelial cellular processes (or proplatelets) to release platelets into the flowing sinusoidal blood. Under inflammatory conditions or acute platelet needs, platelet release can also occur *via* MK rupture (32). In both cases (proplatelet formation and MK rupture), MKs must reside next to the sinusoids to release platelets into the bloodstream.

The MK-EC interactions are regulated by chemokines [e.g., CXCL12 (20, 33–36), FGF4 (20, 37, 38)], adhesion molecules [e.g., VE-cadherin (20), VCAM-1 (20, 39), PECAM-1 (40, 41)], and blood lipids (e.g., sphingosine-1-phosphate or S1P) (42), through many MK cell surface receptors (e.g., CXCR4, a_IIb_b_3_, VLA-4, S1P receptor) (20, 34, 43). ECs have an important role in the regulation of MK maturation and release of platelets (20, 33, 42). A functional vascular niche is critical for platelet production; disrupting the marrow vascular niche (e.g., by inhibiting VE-cadherin which supports EC integrity) impairs MK maturation and thrombopoiesis, while enhancing MK-EC interaction (e.g., by administering CXCL12 or FGF4) increases platelet production (20). (**Table 1**)

**Table 1:** Summary of locally acting factors in marrow microenvironment that modulate megakaryocyte-endothelial cell interactions.

Factor	Effects on MK-EC interactions	References
CXCL12	Promotes the interactions of MKs with the BMECs and the transendothelial migration of MKs *in vivo.*	(20, 33–36)
FGF4	Promotes MK maturation and the adhesion of MKs to ECs both *in vitro* and *in vivo*.	(20, 37, 38)
VE-cadherin	Supports the integrity and formation of BMECs. Neutralizing antibodies to VE-cadherin block FGF4-mediated MK adhesion to BMECs and CXCL12-induced MK transendothelial migration *in vivo*.	(20)
VEGF-A	Promotes angiogenesis and regulates vascular permeability/integrity.	(44, 45)
VCAM-1	Supports the attachment of MKs to HUVECs *in vitro*. Antibodies to VCAM-1 inhibit FGF4-mediated MK adhesion to BMECs and CXCL12-induced MK transendothelial migration *in vivo*.	(20, 39)
PECAM-1	Regulates MK migration towards the vascular niche through modulating the CXCR4 receptor and adhesion molecules (e.g., αIIbβ3) of MKs.	(40, 41)
Thrombospondin	An anti-angiogenic regulator that inhibits marrow vascular regeneration following myelosuppression and inhibits thrombopoiesis.	(46, 47)

MKs also regulate vascular EC function, mediated by the numerous cytokines and growth factors produced by these MK cells. MKs are important sources for both the pro-angiogenic factors such as vascular endothelial growth factor (VEGF) (44, 45) and fibroblast growth factors (FGFs) (6, 48), and the anti-angiogenic factors such as thrombospondin (46) and platelet factor 4 (49). Italiano and colleagues reported that these pro- and anti-angiogenic factors are stored in different alpha-granules and can undergo selective release upon different stimulus (50), providing a mechanism by which MK can differentially stimulate or inhibit the vascular niche under different physiologic and pathologic conditions. These MK vascular regulatory factors may be a key determinant of marrow vascular niche function. However, despite recent progress in understanding the unique process of megakaryocyte lineage development (51–53) and the close interactions between MKs and vascular ECs in the marrow (19, 29) and lung (18, 54), few studies have examined the effects of MKs on vascular niche function and their contributions to normal and neoplastic hematopoiesis.

## Altered MK-Vascular Niche Interactions in Myeloproliferative Neoplasms

In addition to normal cell physiologic studies, the mechanisms by which MKs regulate the vascular niche function can be better understood by assessing the dysregulated MK-EC interactions found in neoplastic hematopoiesis. The myeloproliferative neoplasms (MPNs), which include polycythemia vera (PV), essential thrombocythemia (ET) and primary myelofibrosis (PMF), are stem cell disorders characterized by hematopoietic stem/progenitor cell expansion and overproduction of mature blood cells. Patients with MPNs are often characterized by increased marrow angiogenesis (55–57) and MK hyperplasia (58) when compared to normal marrow. In this section, we will discuss the dysregulated MK-EC interactions and their contributions to the neoplastic hematopoiesis in MPNs.

### Megakaryocytes Are an Important Component of the Perivascular Stem Cell Niche in MPNs

MK hyperplasia is a hallmark feature of MPNs (58) and many MPN-associated genetic mutations/deregulations are preferentially enriched in MKs (59–61). The acquired kinase mutation JAK2V617F plays a central role in MPNs, having been found in virtually all patients with PV and about half of the patients with ET or PMF. To study the effects of a JAK2V617F-bearing MK niche on MPN disease development *in vivo*, we crossed mice that bear a Cre-inducible human JAK2V617F transgene (termed Flip-Flop, or FF1) (62) with the Pf4-cre mice [which bear a Cre recombinase driven by the MK-specific platelet factor 4 promoter (63)] to express JAK2V617F specifically in MKs (Pf4^+^FF1^+^). We found that the JAK2V617F mutant MKs promote the development of an ET phenotype — at 6-mo of age, JAK2V617F transgenic mice manifest modest thrombocytosis, splenomegaly, 2-3 fold increased numbers of marrow MKs, and 2-3 fold increased numbers of marrow HSCs compared to control mice (64). We observed a strong correlation between marrow MK cell numbers and HSC cell numbers in the Pf4^+^FF1^+^ mice, and marrow HSCs in the JAK2V617F-bearing mice are more quiescent and display increased engraftment capacity (65). These results indicate that both HSC number and function are increased in the JAK2V617F-bearing MK niche.

We found that JAK2V617F-driven MK hyperplasia is accompanied by changes in the vascular niche: not only are marrow sinusoids more dilated and MKs are more preferentially located near the marrow sinusoids, but also there is an increased sinusoid vascular density in the Pf4^+^FF1^+^ mice compared to age-matched control mice (64). The effect of JAK2V617F MKs on vascular niche function was studied using tube formation assay (as a measure of *in vitro* angiogenesis) and scratch assay (as a measure of *in vitro* EC migration) — we found that conditioned medium from the JAK2V617F mutant MK culture significantly stimulated EC tube formation and EC migration compared to conditioned medium from wild-type MKs (64). These findings suggest that JAK2V617F mutant MKs can expand the marrow sinusoidal vascular niche, which in turn could contribute to the thrombocytosis and HSC expansion phenotype.

### The JAK2V617F Mutation Alters Megakaryocyte-Endothelial Cell Interactions in the Vascular Niche

**Figure 1:**
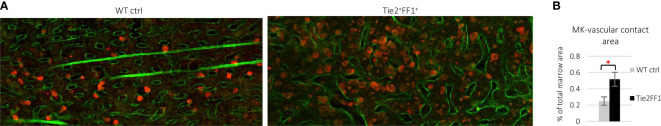
Immunofluorescence staining and deep confocal imaging of marrow MK-EC interactions. **(A)** Representative confocal images of wild-type control (left) and Tie2^+^FF1^+^ (right) mouse femur marrow stained with antibodies against CD41 (red) and laminin (green). **(B)** Quantitative analysis revealed increased area of contact between MKs and sinusoid vessels in the marrow of Tie2^+^FF1^+^ mice compared to the control mice. Two control mice and two Tie2^+^FF1^+^ mice were used and a total of 10 high-quality non-overlapping areas at 20x magnification were selected for analysis with the ImageJ software (National Institute of Health, Bethesda, MD, USA). * P < 0.05.

In addition to mutant blood cells (including MKs), the JAK2V617F mutation is also present in isolated liver, spleen, and marrow ECs from patients with MPNs (66–68). To study the effects of the JAK2V617F mutation on vascular niche function, we employed a murine model in which mice that bear the Cre-inducible human JAK2V617F transgene (FF1) (62) were crossed with a Tie2-Cre transgenic mouse (69) to express JAK2V617F in all hematopoietic cells (including HSCs and MKs) and ECs (Tie2^+^FF1^+^) (70–72). As expected, these mice developed a robust MPN phenotype characterized by neutrophilia, thrombocytosis, splenomegaly, and hematopoietic stem/progenitor cell (HSPC) expansion within 2 months of birth. Histological examination of marrow hematoxylin/eosin sections showed that, compared to control mice, there are markedly increased numbers of MKs in the Tie2^+^FF1^+^ mice and many clusters of MKs are preferentially located near marrow sinusoidal vessels (70). *Ex vivo* co-culture assays revealed that not only do JAK2V617F-bearing ECs directly stimulate JAK2V617F mutant MK expansion, which in turn contributes to HSPC expansion, but also that JAK2V617F mutant MK-conditioned medium significantly stimulates JAK2V617F EC migration compared to that of wild-type MK-conditioned medium. Immunofluorescence staining and deep confocal imaging revealed that marrow MK-EC contact is significantly increased in the Tie2^+^FF1^+^ mice compared to WT control mice (**Figure 1**). These data indicate that the JAK2V617F mutation can alter the MK-EC interactions in the vascular niche to promote the neoplastic hematopoiesis in MPNs.

### Megakaryocyte Niche Function Evolves During Hematopoietic Aging in MPNs, and This is Associated With Changes in its Regulation of the Vascular Niche

Aging within the HSC compartment contributes to many age-related diseases including an increased incidence of hematological malignancies in the elderly. HSC aging is characterized by an expansion of phenotypically defined HSCs with impaired function, such as reduced engraftment and self-renewal capacity, a perturbed state of quiescence, and a skewed differentiation towards the myeloid lineage (73, 74). Studies over the past decade suggest that HSC aging is driven by both cell-intrinsic alterations in the stem cells (75–79), and cell-extrinsic mediators from the aged microenvironment in which the stem cells reside (80–83). The relative contribution of intrinsic and extrinsic mechanisms to HSC aging remains debated. One key question is whether microenvironmental alterations initiate HSC aging or whether aged HSCs cause niche remodeling.

**Figure 2:**
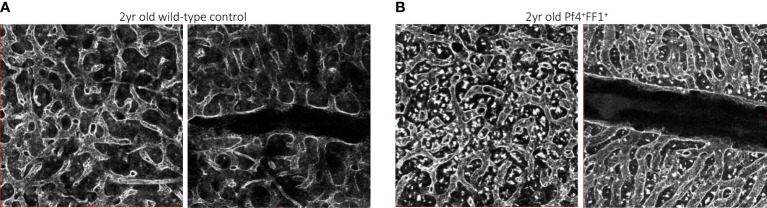
Representative whole-mount confocal images of aged wild-type control **(A)** and Pf4^+^FF1^+^ **(B)** mouse femur marrow, in which the vasculature was stained intravenously with anti-VE-cadherin antibody before euthanization. Images were acquired with an Olympus IX81 microscope using 20x objective magnification and Olympus Fluoview FV1000 confocal laser scanning system at 512 x 512 pixel resolution.

In contrast to the non-hematopoietic niche cells, niche MKs provide direct feedback to their HSC precursors, many of which are located directly adjacent to MKs *in vivo* (8, 9). We investigated the effects of a JAK2V617F-bearing MK niche on HSC aging using the same Pf4^+^FF1^+^ murine model in which the JAK2V617F mutation is expressed exclusively in the MK lineage (64, 84). The mice maintain an essential thrombocythemia phenotype during a 2-yr follow-up, with no evidence of transformation to leukemia or myelofibrosis. Compared to age-matched control mice, the Pf4^+^FF1^+^ mice demonstrated an acceleration of several hallmarks of HSC aging, including an increase in the absolute numbers of HSCs with myeloid-skewed hematopoiesis (and maintenance of this myeloid skewing during marrow transplantation), a reduced engraftment and self-renewal capacity, and a reduced differentiation capacity (84). We showed that the JAK2V617F mutant MK niche can promote hematopoietic aging mediated by enhanced HSC proliferation/cycling. Both flow cytometry analysis and confocal whole-mount imaging revealed decreased marrow EC numbers (especially CD45^-^CD31^+^Sca1^-^ sinusoidal marrow ECs) and decreased marrow vascular areas in 2yr old Pf4^+^FF1^+^ compared to age-matched control mice (84). We noticed altered morphology of the marrow sinusoids in the aged Pf4^+^FF1^+^ mice, which are narrower in diameter compared to marrow sinusoids in aged control mice (**Figure 2**) (84). Conditioned medium collected from JAK2V617F mutant MKs of aged Pf4^+^FF1^+^ mice significantly inhibited EC tube formation *in vitro* (84), which differs from what we have observed in the young (6-mo old) Pf4^+^FF1^+^ mice (64). While the pro-inflammatory factors [IL-6 (85–87), IL-12 (88), MIP-1a (89)] and anti-angiogenic factors [Fas ligand, IL-10 (90, 91)] are upregulated in old JAK2V617F mutant MKs compared to young mutant MKs, their levels are downregulated in aged control MKs compared to young control MKs. These data suggest that the JAK2V617F-bearing MKs inhibit/disrupt the vascular niche during aging, which in turn can promote HSC aging.

Despite recent technical breakthroughs in both imaging and functional studies of HSCs in the marrow, we know very little about how the niche changes with age. Our studies showed that the effect of JAK2V617F mutant MKs on HSC function changes during aging in a murine model of MPN: in young mice, mutant MKs expand the marrow sinusoidal vascular niche, induce HSC quiescence with increased repopulating capacity (64, 65); in aged mice, mutant MKs inhibit/disrupt the vascular niche, promote HSC proliferation with a reduced engraftment and self-renewal capacity (84). Despite these changes in MK niche function during aging, how MKs regulate vascular niche function appears to correlate with how MKs regulate HSC function.

## Concluding Remarks

Further work is needed to functionally test the importance of MK-EC interaction in niche/HSC function and to understand the molecular mechanisms that mediate these effects. Murine models remain an irreplaceable approach to investigating MK-EC interaction *in vivo* in both normal and neoplastic hematopoiesis. Patient-derived induced pluripotent stem cells (iPS) provide an important alternative to model human disease *in vitro*. The capability to differentiate these iPS cell lines towards a specific cell lineage [e.g., MK (92, 93), EC (94)] is a powerful tool to model human diseases with acquired genetic alterations such as the JAK2V617F mutation. It is hoped that such studies will lead to novel therapeutic strategies to target the HSC niche-forming MKs and their interactions with the vascular niche, and to provide better treatments for patients with various aging and neoplastic conditions.

## Author Contributions

HZ wrote the manuscript; KK reviewed and revised the manuscript. All authors contributed to the article and approved the submitted version.

## Funding

This research was supported by the National Heart, Lung, and Blood Institute grant NIH R01 HL134970 (HZ) and VA Merit Award BX003947 (HZ).

## Conflict of Interest

The authors declare that the research was conducted in the absence of any commercial or financial relationships that could be construed as a potential conflict of interest.

## Publisher’s Note

All claims expressed in this article are solely those of the authors and do not necessarily represent those of their affiliated organizations, or those of the publisher, the editors and the reviewers. Any product that may be evaluated in this article, or claim that may be made by its manufacturer, is not guaranteed or endorsed by the publisher.
